# Shuang-Huang-Lian Attenuates Lipopolysaccharide-Induced Acute Lung Injury in Mice Involving Anti-Inflammatory and Antioxidative Activities

**DOI:** 10.1155/2015/283939

**Published:** 2015-04-06

**Authors:** Lei Fang, Yuan Gao, Fen Liu, Rui Hou, Run-Lan Cai, Yun Qi

**Affiliations:** Institute of Medicinal Plant Development, Chinese Academy of Medical Sciences and Peking Union Medical College, Beijing 100193, China

## Abstract

Shuang-Huang-Lian (SHL) is a common traditional Chinese preparation extracted from *Lonicerae Japonicae Flos, Scutellariae Radix*, and *Fructus Forsythiae*. In this study, we demonstrate the anti-inflammatory and antioxidative effects of SHL on lipopolysaccharide- (LPS-) induced acute lung injury (ALI) in mice. SHL reduced the lung wet/dry weight ratio, lowered the number of total cells in the bronchoalveolar lavage fluid, and decreased the myeloperoxidase activity in lung tissues 6 h after LPS treatment. It also inhibited the overproduction of proinflammatory cytokines (TNF-*α*, IL-1*β*, and IL-6) in the bronchoalveolar lavage fluid. Histological studies demonstrated that SHL attenuated LPS-induced interstitial edema, hemorrhage, and the infiltration of neutrophils into the lung tissue. Moreover, SHL could also enhance the superoxide dismutase and catalase activities, increase the reduced glutathione content, and decrease the malondialdehyde content. The present results suggest that SHL possesses anti-inflammatory and antioxidative properties that may protect mice against LPS-induced ALI.

## 1. Introduction

Acute lung injury (ALI), a common clinical complication of microbial infections, is the major cause of respiratory failure [1]. It is characterized by intense parenchymal inflammatory processes, including neutrophil influx into the alveolar space and the release of proinflammatory cytokines, such as tumor necrosis factor-*α* (TNF-*α*), interleukin-1*β* (IL-1*β*), and interleukin-6 (IL-6). The excessive accumulation of inflammatory mediators contributes to increasing the permeability of the alveolar-capillary barrier, interstitial protein-rich edema, hemorrhage, and the subsequent development of respiratory distress syndrome [2, 3]. Furthermore, the overproduction of reactive oxygen species (ROS) during ALI amplifies cellular damage, which involves the lipid peroxidation of membrane phospholipids, affects antioxidant enzymes activities, including superoxide dismutase (SOD) and catalase (CAT), and depletes sulfhydryl-bearing molecules, such as glutathione (GSH) [4, 5]. Despite substantial insights into the pathogenesis of ALI, an effective therapy for ALI remains sought after, and the mortality of this disease remains high [6].

Shuang-Huang-Lian (SHL), a common traditional Chinese preparation, is extracted from* Lonicerae Japonicae Flos, Scutellariae Radix,* and* Fructus Forsythiae*. Clinically, SHL products are administered via different routes (e.g., oral, injectable, pulmonary, and rectal routes) and used as antimicrobial agents to treat various acute respiratory infections [7]. However, the antibacterial power of SHL is very weak compared with other antibacterial agents. Therefore, SHL has been mainly used as an antiviral agent in China [8]. Some of the major chemical ingredients in SHL, such as chlorogenic acid, baicalin, and forsythiaside, have previously been reported to possess both anti-inflammatory and antioxidative activities. Moreover, our recent* in vitro* study also revealed that SHL alleviated the oxidative burden by decreasing the intracellular ROS levels and inhibited the production of inflammatory mediators by suppressing the p38- and ERK1/2-mediated AP-1 pathway in LPS-activated murine alveolar macrophages [9]. Alveolar macrophage activation is known to be a minor local response during the pathological process of ALI. Unlike* in vitro* studies,* in vivo* studies provide more persuasive conclusions for systemic responses, which are relevant to clinical applications. In the present study, we explore the* in vivo* effects of SHL in LPS-induced ALI mice.

## 2. Materials and Methods

### 2.1. Materials and Reagents

Injectable SHL was purchased from Duoduo Pharmaceutical Co., Ltd. (Jiamusi, Heilongjiang, China). Mouse TNF-*α*, IL-1*β*, and IL-6 ELISA kits were obtained from Excell Technology Co. (Shanghai, China). SOD was purchased from the Beyotime Institute of Biotechnology (Jiangsu, Haimen, China). 3, 3′, 5, 5′-Tetramethylbenzidine (TMB) was purchased from Sangon Biotech Co. (Shanghai, China). Dexamethasone (DXM), lipopolysaccharide (*Escherichia coli* O55:B5, LPS), GSH, 4-hydroxy-TEMPOL (TEM), and 1, 1, 3, 3-tetraethoxypropane (TEP) were purchased from Sigma-Aldrich (St. Louis, MO, USA). All other reagents were of analytical grade.

### 2.2. Identification of SHL Constituents

In our previous study, we identified the main components in SHL using HPLC-UV analysis. The major components include baicalin, forsythiaside B, forsythiaside A, and rutin (1,078.23 *μ*g, 47.85 *μ*g, 40.97 *μ*g, and 13.93 *μ*g in 1 mL of SHL, resp.) [9].

### 2.3. Animals and Treatment

**Figure 1:**
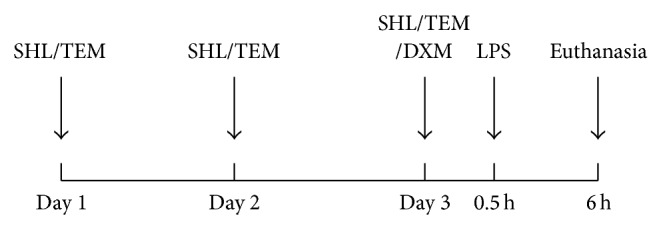
Schedule of pretreatment with SHL and positive drugs and mice induced by LPS.

Balb/c mice (male, 18–20 g) were purchased from Vital River Experimental Animal Services (Beijing, China) and housed in standard laboratory temperature and humidity conditions with a 12 h light/dark cycle. They were randomly divided into five groups: normal control group (NC), model group (LPS), SHL groups (5 and 10 mL/kg), and positive control group (DXM or TEM). Because glucocorticoids have widely been used as anti-inflammatory drugs in the clinical treatment of ALI and TEM has also been shown to act as a SOD mimetic to significantly protect from LPS-induced ALI [10], we selected DXM and TEM as positive controls to evaluate the anti-inflammatory and antioxidative effects of SHL. The pretreatment with SHL and positive drugs as a function of time is shown in Figure 1; SHL or TEM was administered intraperitoneally (i.p.) once per day for 3 consecutive days, while DXM was administered once 30 min prior to LPS administration. Mice in the control and model groups received an equal volume of physiological saline. The mice were anesthetized 30 min after the last administration. Subsequently, LPS (2 *μ*g per mouse) was intratracheally instilled [11]. The mice in the control group received an equal volume of physiological saline without LPS.

Three independent experimental parameters were tracked: (1) the lung wet/dry weight ratio was measured; (2) the bronchoalveolar lavage fluid (BALF) was collected for cell counting and cytokine assays; and (3) pathological studies and lung antioxidant enzymes assays were conducted. All experiments were carried out according to the National Institutes of Health Guide for the Care and Use of Laboratory Animals and were approved by the Animal Ethics Committee of the Institute of Medicinal Plant Development, Chinese Academy of Medical Sciences.

### 2.4. Lung Wet/Dry Weight (*W/D*) Ratio

The mice were sacrificed and lungs were excised 6 h after LPS challenge. Any blood was removed by blotting the lungs with filter papers until dry, and the lungs were then immediately weighed. The lungs were subsequently placed in an oven at 60°C until the weight ceased to change, and the dry weight was recorded. The lung* W/D* ratio was calculated to assess tissue edema.

### 2.5. Collection of BALF

BALF was collected as previously described [12]. Briefly, mice were sacrificed 6 h after LPS treatment. The BALF was obtained by intratracheal intubation. The lungs were lavaged three times with 0.5 mL of physiological saline to collect a total of approximately 1.3 mL of BALF. The BALF from each sample was centrifuged (4°C, 500 ×g, 15 min), and the supernatants were stored at −70°C for the subsequent analysis of cytokine production. The erythrocytes were removed from the resuspensions via the addition of 0.8% ammonium chloride solution for 1 min, followed by two washes. The cells were then resuspended to the original volume in PBS. The cells were counted with a hemocytometer.

### 2.6. ELISA for TNF-*α*, IL-1*β*, and IL-6 in BALF

TNF-*α*, IL-6, and IL-1*β* in BALF were assayed using ELISA kits according to the manufacturer's instructions. The concentrations were calculated based on standard curves.

### 2.7. Pathological Studies

The whole lungs were harvested and fixed in 10% neutral-buffered formalinfor 24 h. The lungs were histologically processed, embedded in paraffin, sectioned on a microtome, and stained with hematoxylin and eosin (H&E). The histopathology was observed under a light microscope.

To analyze leukocyte recruitment, the number of macrophages was adjusted to 1 × 10^5^ cells/mL in BALF with a cytocentrifuge, and the leukocytes on the H&E-stained slide were observed.

### 2.8. Preparation of Tissue Homogenates

At the termination of the experiment, the mice were sacrificed, and the lungs were excised. After being blotted dry on filter papers, the left lungs were weighed and homogenized in PBS using automatic tissue homogenizer. The homogenate was then centrifuged at 3,000 rpm for 10 min at 4°C. The resultant supernatants were used to assess the SOD and CAT activities as well as the GSH content. The remaining pellets were resuspended in extraction buffer (50 mM phosphate buffer containing 0.5% cetyltrimethyl ammonium bromide, pH 6.0) and homogenized again. The homogenate was centrifuged at 10,000 ×g for 15 min at 4°C. The myeloperoxidase (MPO) activity in the supernatants was assessed. The right lungs were weighed and homogenized in 0.15 M KCl, and the supernatants were used for the lipid peroxidation assay.

### 2.9. SOD Activity Assays

The activity of SOD was determined based on the inhibition of nitrite production by SOD due to the superoxide anion generated in a xanthine-xanthine oxidase system, as previously described [9]. The activity of SOD is expressed as U/mg of protein. The protein concentrations in the lung homogenates were quantified using the Bradford method [13].

### 2.10. CAT Activity Assays

The activity of CAT was measured as previously described [14]. An aliquot (0.2 mL) of supernatant was mixed with 1 mL of 65 *μ*M H_2_O_2_ at 37°C for 1 min, and 1 mL of 32.4 mM ammonium molybdate was then immediately added, and the absorbance of the reaction mixture was read at 405 nm. The protein concentrations in the lung homogenates were quantified using the Bradford method [13]. The activity of CAT is expressed as U/mg of protein.

### 2.11. Lipid Peroxidation Assays

The lipid peroxidation was measured with a thiobarbituric acid reactive substances (TBARS) assay as previously described [15]. The TBARS results are expressed as malondialdehyde (MDA) equivalents using TEP as a standard.

### 2.12. GSH Content Assay

The GSH content was measured as previously described [8]. Briefly, the protein concentrations were quantified, and the lung homogenates were then deproteinized with 0.5 M HClO_4_ for 2 min, followed by neutralization with 1 M K_2_CO_3_ and centrifugation at 10,000 ×g for 15 min at 4°C. The supernatants (50 *μ*L) were transferred to 96-well black flat-bottom plates, and 180 *μ*L of PBS and 50 *μ*L of *ο*-phthalaldehyde (1 mg/mL in methanol) were then added. The fluorescence was measured at an excitation wavelength of 355 nm and an emission wavelength of 405 nm using a fluorescence microplate reader. The GSH content, in nmol/mg of protein, was calculated from the GSH calibration curve.

### 2.13. MPO Activity Assays

The MPO activity was measured according to a method by Queiroz et al. [16]. Briefly, the supernatant containing MPO was added to the assay buffer (0.5 mM H_2_O_2_, 1.4 mM TMB, and 8% dimethylformamide in 50 mM acetate buffer, pH 5.4). The mixture was incubated at 37°C for 5 min. The absorbance, which was indicative of the TMB oxidation by MPO, was measured at 655 nm. The enzyme activity was calculated as units per milligram of protein. The protein concentrations were quantified using Bradford's method [13].

### 2.14. Statistical Analysis

The data are presented as the mean ± SD and were analyzed using a one-way ANOVA. Student's *t*-test was used when only two groups were compared. Differences were considered to be significant at *P* < 0.05.

## 3. Results

### 3.1. Effect of SHL on Lung* W/D* Ratio

**Figure 2:**
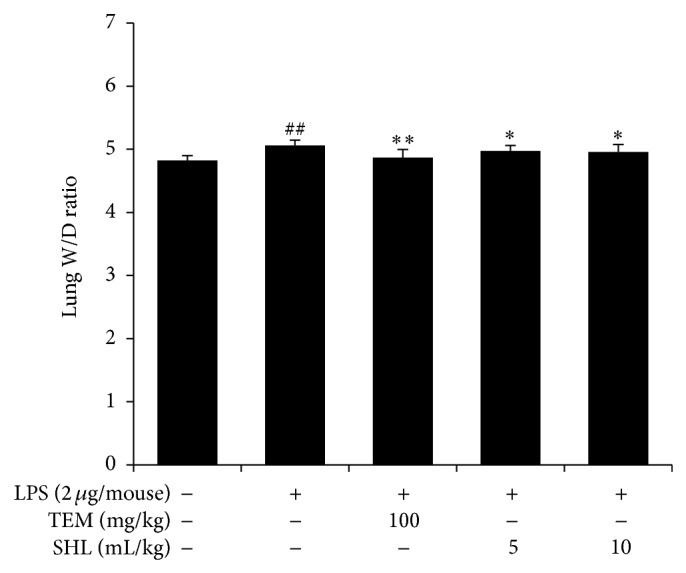
Effects of SHL on the lung* W/D* ratio in LPS-induced ALI mice. Mice received (i.p.) SHL or TEM once daily for 3 days. The lung* W/D* ratio was determined 6 h after LPS application. The data represent means ± SD (*n* = 10). ^##^ *P* < 0.01 versus control group; ^*^ *P* < 0.05, ^**^ *P* < 0.01 versus LPS group.

The lung* W/D* ratio was evaluated 6 h after LPS challenge. The results (Figure 2) showed an increase in the lung* W/D* ratio in the ALI group compared with the control group (*P* < 0.01). Pretreatment with SHL or TEM lowered the lung* W/D* ratio (*P* < 0.05), suggesting that SHL could attenuate pulmonary edema.

### 3.2. Effect of SHL on Histopathological Change in Lung Tissues

**Figure 3:**
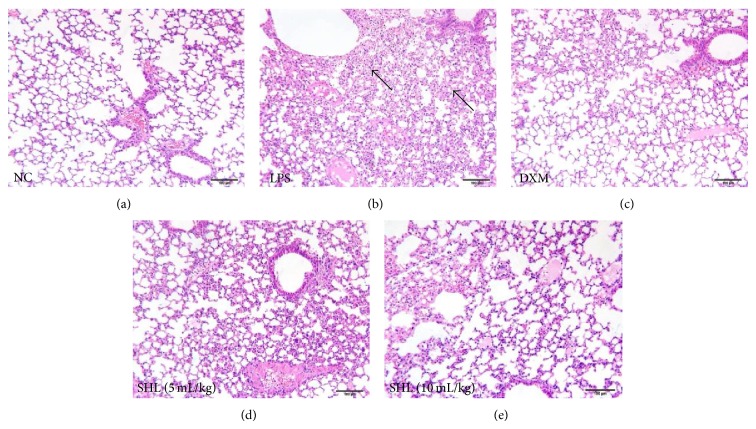
Effect of SHL on histopathological change in lung tissues (200x, H&E staining). Mice received (i.p.) SHL (5 and 10 mL/kg) once daily for 3 days or DXM (5 mg/kg) 30 min before LPS administration. Lungs (*n* = 3) were processed to assess histological changes 6 h after LPS challenge. The arrows point to prominent neutrophil infiltration.

A normal pulmonary structure was observed in the control mice (Figure 3(a)). In contrast, LPS-treated animals exhibited a moderate inflammatory reaction in the lung characterized by interstitial edema, alveolar hemorrhage, and prominent neutrophil infiltration into the interstitium and alveolar spaces (Figure 3(b)). Mice treated with SHL (10 mL/kg) or DXM showed a minimal inflammatory reaction manifested by minor interstitial edema and fewer neutrophils that invaded into the interstitium and alveolar spaces (Figures 3(c)–3(e)).

### 3.3. Effect of SHL on TNF-*α*, IL-6, and IL-1*β* Production in BALF

**Figure 4:**
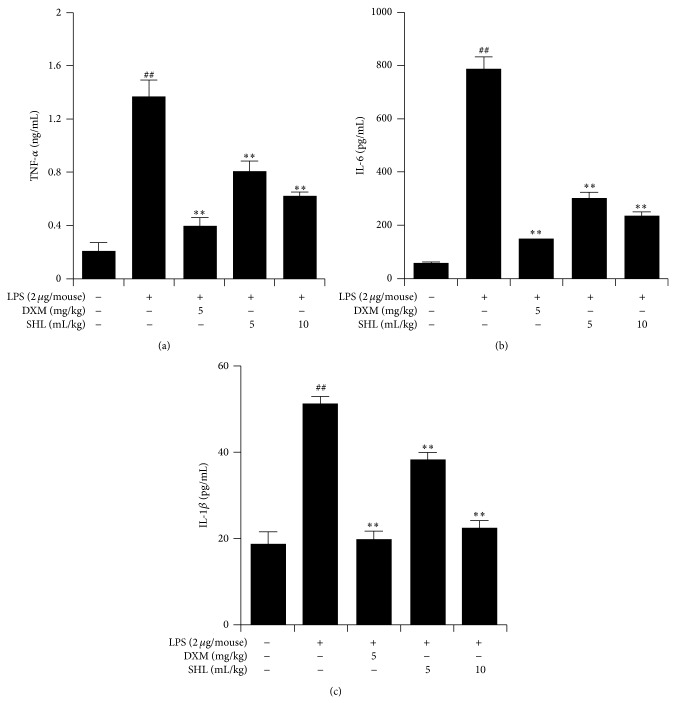
Effect of SHL on production of inflammatory cytokines (TNF-*α*, IL-1*β*, and IL-6) in BALF of LPS-induced ALI mice. Mice received (i.p.) SHL once daily for 3 days or DXM 30 min before LPS administration. BALF was collected 6 h after LPS administration to analyze TNF-*α* (a), IL-6 (b), and IL-1*β* (c) production. Data present the means ± SD (*n* = 10). ^##^ *P* < 0.01 versus control group; ^**^ *P* < 0.01 versus LPS group.

The effect of SHL on proinflammatory cytokines production was examined. As shown in Figure 4, exposure to LPS resulted in a significant increase (*P* < 0.01) in TNF-*α*, IL-1*β*, and IL-6 production compared with the control group. Pretreatment with SHL or DXM efficiently inhibited the TNF-*α*, IL-1*β*, and IL-6 production in a dose-dependent manner (*P* < 0.01).

### 3.4. Effect of SHL on Leukocytes in BALF

**Figure 5:**
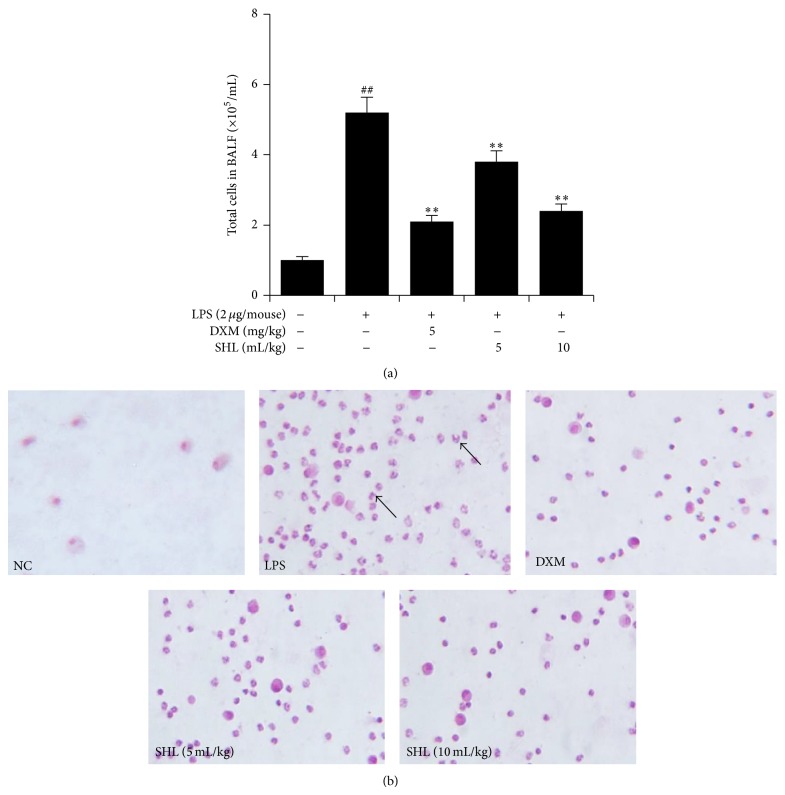
Effect of SHL on leukocyte recruitment in BALF. Mice received (i.p.) SHL (5 and 10 mL/kg) once daily for 3 days or DXM (5 mg/kg) 30 min before LPS administration. BALF was collected 6 h after LPS application, and cells were stained by H&E. (a) Total cell number in BALF. (b) Leukocyte recruitment in BALF from mouse lungs (400x). The arrows point to leukocytes. Data represent means ± SD (*n* = 3). ^##^ *P* < 0.01 versus control group; ^*^ *P* < 0.05, ^**^ *P* < 0.01 versus LPS group.

As shown in Figure 5(a), the number of total cells in the BALF dramatically increased after LPS application compared with the control mice (*P* < 0.01). SHL markedly decreased the number of total cells in a dose-dependent manner (*P* < 0.01).

The number of macrophages in the BALF was adjusted to 1 × 10^5^ cells/mL and the fluid was spun a cytocentrifuge to observe the leukocytes. As shown in Figure 5(b), the number of leukocytes in the BALF was significantly higher after LPS treatment. The administration of SHL or DXM effectively decreased the number of leukocytes in the BALF.

### 3.5. Effect of SHL on the MPO Activity in Lung Tissues

**Figure 6:**
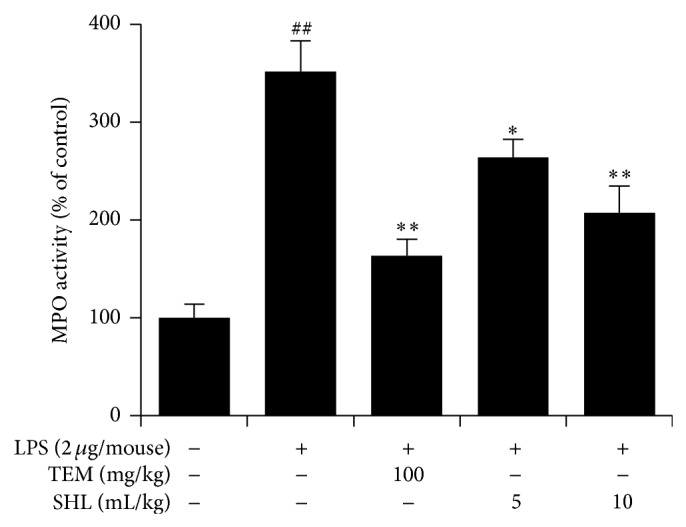
Effects of SHL on MPO activity. Mice received SHL or TEM once daily for 3 days. Lung homogenates were prepared 6 h after LPS exposure, and the activity of MPO was determined. Data represent means ± SD (*n* = 10). ^##^ *P* < 0.01 versus control group; ^*^ *P* < 0.05, ^**^ *P* < 0.01 versus LPS group.

MPO, an enzyme located mainly in the primary granules of neutrophils, reflects the adhesion and margination of neutrophils during the pathogenesis of ALI [17]. The MPO activity in the lung tissue was measured to confirm the effect of SHL on neutrophil accumulation. As shown in Figure 6, LPS application increased the MPO activity 3.6-fold. Pretreatment with SHL reduced the rate of TMB oxidation by MPO in a dose-dependent manner, indicating that SHL could alleviate the infiltration of neutrophils, which are rich in MPO. These results were consistent with the effect of SHL on neutrophil recruitment in the BALF.

### 3.6. Effect of SHL on Oxidative Stress

**Figure 7:**
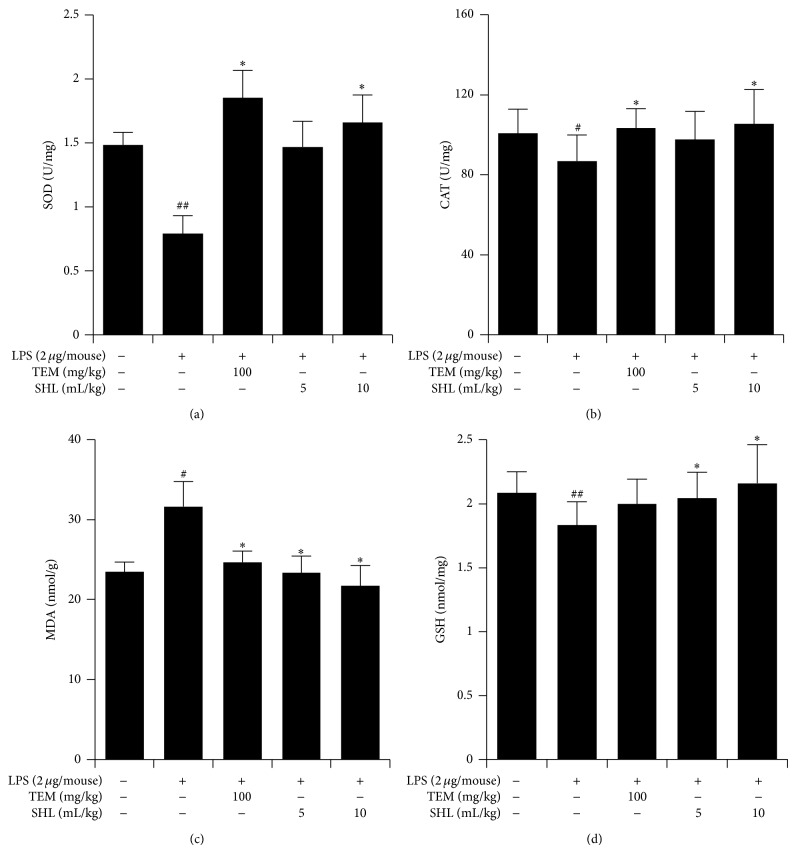
Effects of SHL on the activities of SOD and CAT and the content of GSH and MDA in lung tissues. Mice received SHL or TEM once daily for 3 days. Lung homogenates were prepared 6 h after LPS treatment. Activities of SOD (a) and CAT (b) and the content of MDA (c) and GSH (d) were determined. Data represent means ± SD (*n* = 10). ^#^ *P* < 0.05 and ^##^ *P* < 0.01 versus control group; ^*^ *P* < 0.05 and ^**^ *P* < 0.01 versus LPS group.

Oxidative stress plays an important role in the development of LPS-induced ALI. To evaluate the effects of SHL on oxidative stress, the activities of SOD and CAT as well as the contents of GSH and MDA in the lungs were determined. As shown in Figures 7(a)–7(c), LPS markedly reduced the SOD (*P* < 0.01) and CAT activities (*P* < 0.05) and induced an overproduction of MDA (*P* < 0.05). SHL (5 and 10 mL/kg) or TEM could decrease the MDA content (*P* < 0.05), and 10 mL/kg SHL increased the SOD and CAT activities (*P* < 0.05).

The depletion of GSH, and essential intra- and extracellular protective antioxidant, by LPS is an important event that contributes to the increase in the ROS level [18]. The GSH levels are significantly reduced in the BALF ALI patients [19]. Similarly, we found that the GSH content also significantly decreased in the ALI mice (*P* < 0.01) compared with the normal group. Pretreatment with SHL increased GSH content compared with the LPS-induced model group (*P* < 0.05) (Figure 7(d)). Taken together, these data indicate that SHL could inhibit oxidative stress in ALI mice.

## 4. Discussion 

In this study, the anti-inflammatory and antioxidative effects of SHL were investigated in LPS-induced ALI mice. Our results show that SHL effectively attenuated the LPS-induced pulmonary edema, infiltration of neutrophils, production of proinflammatory cytokines, and oxidative stress.

LPS, a major component of the outer membranes in Gram-negative bacteria, has been recognized as the most important pathogen in pulmonary inflammation that leads to ALI [17]. The intratracheal instillation of LPS may be more reasonable than intranasal administration to induce ALI in mice [20] because it ensures an accurate dose of LPS in the lungs. Furthermore, this model mimics the critical pathogenesis of ALI, especially the marked accumulation of polymorphonuclear neutrophils and the release of mediators [21]. Moreover, this approach provokes symptoms similar to those observed in human patients [22]. In previous studies, the concentrations TNF-*α*, IL-1*β*, and IL-6 in the BALF peaked 6 h after LPS application [23–25]. Furthermore, tissue injury and an increase in the lung* W/D* ratio could also be assessed at this time [25, 26]. Thus, we investigated the cytokines in the BALF, the lung* W/D* ratio, and morphological changes in mouse tissue samples obtained 6 h after the intratracheal instillation of LPS. Notably, LPS instillation contributes to the local, rather than systemic, release of proinflammatory mediators [27]. In fact, we also determined the TNF-*α* level in the serum of LPS-induced ALI mice at the same time point. In contrast to the levels in the BALF, the serum TNF-*α* did not significantly increase after LPS application (data not shown).

As expected, LPS could increase the lung* W/D* ratio and the number of neutrophils in the BALF to consequently enhance the MPO activity in lung tissues. Similarly, the lung histological examination also confirmed that the infiltration of neutrophils and alveolar edema was prominent in ALI mice. SHL successfully attenuated pulmonary edema and reduced the number of leukocytes in the tissue. Combined with the effects of SHL on MPO, we concluded that SHL could inhibit the recruitment of leukocytes, in particular neutrophils [17]. Furthermore, the overproduction of proinflammatory cytokines derived predominantly from macrophages (e.g., TNF-*α*, IL-1*β*, and IL-6) in the lung is associated with neutrophil activation, capillary leakage, and tissue edema during the development of ALI [28]. Our data show that pretreatment with SHL dose dependently decreased the production of these cytokines, indicating that SHL exerts a significant anti-inflammatory effect on LPS-induced ALI mice.

Under basal conditions, various antioxidative enzymes (e.g., CAT and SOD) and low-molecular weight scavengers (e.g., millimolar levels of GSH) in the lung tissue remove ROS (e.g., O_2_^•−^, H_2_O_2_, and ^•^OH) to preserve normal physiologic functions. SOD converts O_2_^•−^ into H_2_O_2_, which can be catalyzed into H_2_O by CAT [4]. In LPS-induced ALI mice, activated phagocytic cells migrate into tissues and release a variety of excessive ROS. These cytotoxic oxidants lead to the depletion of GSH, inactivation of CAT and SOD, and eventually the lipid peroxidation of membrane phospholipids and formation of MDA [5]. More recently, our* in vitro* study demonstrated that SHL dose dependently decreased the levels of intracellular ROS, enhanced the SOD activity, increased the GSH content, and reduced MDA production in LPS-stimulated murine alveolar macrophages [9]. Similarly, in the present* in vivo* study, SHL effectively increased the activities of lung SOD and CAT and reduced the lung MDA content in LPS-induced ALI mice. These results indicate that SHL exerts a significant antioxidant effect on LPS-induced ALI mice.

In conclusion, we herein further investigated the anti-inflammatory and antioxidative effects of SHL in LPS-induced ALI mice based on our previous* in vitro* study [9]. SHL attenuated pulmonary edema, reduced the recruitment of neutrophils into the lung tissue, and inhibited the production of inflammatory mediators. Moreover, SHL alleviated the oxidative burden by increasing the activities of antioxidant enzymes and micromolecular scavenger content. These data suggest that SHL protects lung tissue from infection-induced injury via its potential anti-inflammatory and antioxidative activities as well as its antimicrobial effect. We believe that the present study will help to expand the clinical application of SHL in bacterium-induced respiratory infections.
